# FGFR-TKI resistance in cancer: current status and perspectives

**DOI:** 10.1186/s13045-021-01040-2

**Published:** 2021-02-10

**Authors:** Sitong Yue, Yukun Li, Xiaojuan Chen, Juan Wang, Meixiang Li, Yongheng Chen, Daichao Wu

**Affiliations:** 1 Department of Oncology, Laboratory of Structural Biology, NHC Key Laboratory of Cancer Proteomics, State Local Joint Engineering Laboratory for Anticancer Drugs, Xiangya Hospital, Central South University, Changsha, 410008 Hunan China; 2 Clinical Anatomy and Reproductive Medicine Application Institute, Department of Histology and Embryology, Hunan Province Key Laboratory of Cancer Cellular and Molecular Pathology, University of South China, Hengyang, 421001 China; 3 National Clinical Research Center for Geriatric Disorders, Xiangya Hospital, Central South University, Changsha Hunan, 410008 China; 4 W.M. Keck Laboratory for Structural Biology, University of Maryland Institute for Bioscience and Biotechnology Research, Rockville, MD 20850 USA

**Keywords:** FGFR, Tyrosine kinase inhibitor, Drug resistance, Gatekeeper mutation, Lysosome sequestration

## Abstract

Fibroblast growth factor receptors (FGFRs) play key roles in promoting the proliferation, differentiation, and migration of cancer cell. Inactivation of FGFRs by tyrosine kinase inhibitors (TKI) has achieved great success in tumor-targeted therapy. However, resistance to FGFR-TKI has become a concern. Here, we review the mechanisms of FGFR-TKI resistance in cancer, including gatekeeper mutations, alternative signaling pathway activation, lysosome-mediated TKI sequestration, and gene fusion. In addition, we summarize strategies to overcome resistance, including developing covalent inhibitors, developing dual-target inhibitors, adopting combination therapy, and targeting lysosomes, which will facilitate the transition to precision medicine and individualized treatment.

## Background

Fibroblast growth factor receptors (FGFRs), a subfamily of receptor tyrosine kinases (RTKs), consist of five members (FGFR1-5) that share remarkable sequence homology [1]. They typically contain the extracellular domain, hydrophobic transmembrane domain, and intracellular tyrosine kinase domain [2, 3]. Unlike the other four members, FGFR5 (known as FGFRL1) lacks a tyrosine kinase domain. It plays a role in regulating excessive activation of the FGF-FGFR1 signaling pathway [4, 5]. The signaling axis of FGFRs is primarily activated in a ligand-dependent manner, by binding of fibroblast growth factors (FGFs) and the subsequent receptor dimerization induced intracellular kinase transautophosphorylation events [6]. Meanwhile, FGFRs can also be activated in a ligand-independent manner, such as chromosome translocation induced FGFRs gene fusion with other constitutively expressed genes [7].

The FGF/FGFR signaling pathway is closely related to the occurrence of embryogenesis, angiogenesis, tissue homeostasis, and wound repair [8]. It also plays critical roles in cell proliferation, differentiation, apoptosis, and migration [9, 10]. Aberrantly activated FGF/FGFR signaling axis leads to a variety of diseases, especially malignant tumors, which are caused by gene amplification, mutation, and gene fusion [11, 12].

Blocking the FGF/FGFR signaling axis by tyrosine kinase inhibitors (TKIs) was proved to be a successful therapeutic strategy in numerous tumor types [13]. Erdafitinib was the first approved FGFR-TKI for treating metastatic urothelial carcinoma based on remarkable results of the phase II trial (Table 1, NCT02355597) [14, 15]. And a further phase III trial (NCT03390504) is being performed to compare the efficacy of Erdafitinib versus Vinflunine or Docetaxel or Pembrolizumab in advanced urothelial cancer. Multiple clinical trials are being conducted on the effectiveness of Erdafitinib in a variety of cancers, such as non-small cell lung cancer (NCT03827850), advanced solid tumor (NCT02465060, NCT03155620, NCT03120714, NCT04083976), breast cancer (NCT03238196), liver cancer (NCT02421185) and castrated prostate cancer (NCT03999515). Subsequently, because of the excellent phase II results (Table 1, NCT02924376), FDA authorized FGFR-TKI Pemigatinib as the first targeted therapy for advanced cholangiocarcinoma in April 2020 [16, 17]. And various indications for Pemigatinib are undergoing clinical trials, such as non-muscle invasive bladder cancer (NCT03914794), solid tumor (NCT03235570, NCT04258527, NCT03822117, NCT04003623), urothelial cancer (NCT04294277, NCT04003610, NCT02872714), acute myeloid leukemia (NCT04659616), myeloproliferative tumor (NCT03011372), colorectal cancer (NCT04096417), lung cancer and gastric cancer (NCT02393248).

**Table 1:** Clinical development of FGFR-TKIs

Drugs	Company	Targets	Approved/clinical trials	Patients and clinical results
Erdafitinib (JNJ-42756493)	Janssen	Pan-FGFR	*FDA approved* Phase II NCT02365597	Advanced or Metastatic urothelial carcinoma with FGFR2/FGFR3 genetic alterations and had a history of disease progression within 12 months of neoadjuvant or adjuvant platinum-containing chemotherapy. Results: ORR: 40%; median PFS: 5.5 months; median OS: 13.8 months
			Phase I/IIa NCT02421185	Advanced hepatocellular carcinoma with FGF19 amplification. Results: ORR: 4.8%; DCR: 35.7% VS 9.1%; median PFS: 1.58 months VS 1.31 months (FGF19 amplification VS no-FGF19 amplification)
Pemigatinib (INCB054828)	Incyte	Pan-FGFR	*FDA approved* Phase II NCT02924376	Documented disease progression following at least one previous systemic cancer therapy, locally advanced or metastatic cholangiocarcinoma with FGFR2 gene fusion or rearrangement. Results: ORR: 35.5%; median PFS: 6.9 months; median OS: 21.1 months; median DOR: 7.5 months; DCR: 82.0%
Futibatinib (TAS-120)	Taiho Pharm	Pan-FGFR	Phase II NCT02052778	Disease progression after ≥ 1 line of systemic therapy (gemcitabine plus platinum-based chemotherapy), advanced or metastatic unresectable intrahepatic cholangiocarcinoma with FGFR2 gene fusions or other rearrangements. Results: ORR: 34.3%; DCR: 76.1%; median DOR: 6.2 months
CH5183284 (Debio-1347)	Debio	FGFR1/2/3	Phase II NCT03834220	Solid tumors harboring FGFR 1/2/3 gene fusion or rearrangement
ASP5878	Astellas	Pan-FGFR	Phase I NCT02038673	Urothelial carcinoma, hepatocellular carcinoma, or squamous cell lung carcinoma with FGFRs genetic alterations
Dovitinib (TKI258)	Novartis	FGFR1/2/3; KIT; VEGFR	Phase II NCT01861197	Advanced squamous non-small cell lung cancer with FGFR1 amplification. Results: ORR: 11.5%; DCR: 50%; median PFS: 2.9 months; median OS: 5.0 months
			Phase II NCT01379534	FGFR2 mutated or WT advanced and/or metastatic endometrial cancer. Results: ORR: 4.5% VS 16.5%; DCR: 63.6% VS 51.6%; PFS: 4.1 months VS 2.7 months; 18-week PFS rate: 31.8% VS 29%; median OS: 20.2 months VS 9.3 months (FGFR2 mutation VS FGFR2 WT)
PRN1371	Principia	Pan-FGFR	Phase I NCT02608125	Metastatic urothelial carcinoma with FGFRs genetic alterations
LY2874455	Eli-Lilly	Pan-FGFR; VEGFR2	Phase I NCT01212107	Advanced solid-organ cancer
Infigratinib (BGJ398)	Novartis	Pan-FGFR	Phase II NCT02150967	Advanced or metastatic cholangiocarcinoma with FGFRs alterations whose disease progressed despite prior systemic therapy. Results: ORR: 14.8%; DCR: 75.4%; median PFS: 5.8 months
			Phase II NCT02160041	Solid tumor and hematologic malignancies with FGFRs genetic alterations. Results: CBR: 15%; ORR: 7.5%; median PFS: 1.8 months; OS: 6.2 months
AZD4547	AstraZeneca	Pan-FGFR	Phase II NCT02465060	Advanced refractory solid tumors, lymphomas, or multiple myeloma with FGFR1/2/3 aberrations. Results: Median PFS: 3.4 months, 6-month PFS rate: 15%; (For FGFR fusions patients, ORR: 22%, 6-month PFS: 56%)
Derazantinib (ARQ-087)	Basilea	Pan-FGFR; RET; DDR2; KIT;VEGFR; PDGFRβ	Phase I/II NCT01752920	Advanced, inoperable, or metastatic solid tumors with FGFRs genetic alterations who failed to respond to standard therapy or for whom standard curative therapy does not exist. Results: ORR: 20.7%; DCR: 82.8%; median PFS: 5.7 months
E7090	Eisai	FGFR1/2/3	Phase II NCT04238715	Unresectable advanced or metastatic cholangiocarcinoma with FGFR2 gene fusions
HMPL-453	Chi-Med	FGFR1/2/3	Phase II NCT04353375	Advanced bile duct cancer with FGFR2 fusions
Rogaratinib (BAY-1163877)	Bayer	Pan-FGFR	Phase III NCT03410693	Advanced or metastatic urothelial carcinoma with FGFR-positive after receiving prior platinum-containing chemotherapy (Rogaratinib VS chemotherapy)
Roblitinib (FGF401)	Novartis	FGFR4	Phase I/II NCT02325739	FGF19-driven hepatocellular cancer
ODM-203	Orion	FGFR; VEGFR1/2/3	Phase I/IIa NCT02264418	Advanced or metastatic solid tumors for which treatment according to the guidelines was no longer available. Results: ORR: 9.2%; median PFS: 16.1 and 12.4 weeks for aberrant or non-aberrant FGFR
ICP-192	InnoCare	Pan-FGFR	Phase II NCT04492293	Surgically unresectable or metastatic bladder urothelial cancer with FGFRs genetic aberrations
H3B-6527	Eisai /H3	FGFR4	Phase I NCT02834780	Advanced hepatocellular carcinoma and intrahepatic cholangiocarcinoma
Fisogatinib (BLU-554)	Blueprint	FGFR4	Phase I NCT02508467	FGF19 positive advanced hepatocellular carcinoma. Results: ORR: 17% VS 0%; median PFS: 3.3 months VS 2.3 months (FGF19-positive VS FGF19-negative)

*ORR* objective response rate, *DCR* disease control rate, *PFS* progression-free survival, *OS* overall survival, *DOR* duration of response, *CBR* clinical benefit rate

In addition to these two approved FGFR-TKIs, numerous candidate FGFR-TKIs are developed and pushed into phase I or II clinical trials (Table 1). They include selective FGFR inhibitors, multi-target kinase inhibitors, covalent FGFR inhibitors, and FGFR4 specific inhibitors. And some of them have achieved significant progress based on clinical trials. For example, BGJ398 has been granted Fast Track Designation by FDA for treating cholangiocarcinoma with FGFR2 gene fusions due to the phase II study (NCT02150967) with an encouraging progression-free survival (PFS, 5.8 months), response rate (14.8%), and disease control rate (75.4%) [18]. By contrast, AZD4547 did not obviously improve PFS versus paclitaxel in gastric cancer patients harboring FGFR2 amplification [19].

Although the FGFR-TKIs have shown promising results in targeted therapies, resistance to FGFR-TKIs is becoming increasingly prominent. Here, we summarize the mechanism of FGFR-TKI resistance in cancer, and provide reasonable perspectives to overcome this resistance.

## Mutations in kinase, especially at gatekeeper residues, confer resistance to FGFR-TKI

In a study of next-generation sequencing technology based on more than 4,000 tumors, FGFRs mutations account for around 26% of cancers with FGFRs gene abnormality [20]. FGFRs kinase mutations are the most common mechanism of FGFR-TKI resistance in targeted therapy. We categorize these mutations into gatekeeper mutations and other mutations. Gatekeeper residues are located in the Hinge region of the ATP-binding pocket of kinases. They play a role in controlling access of TKIs to the hydrophobic ATP binding pocket and advance active conformation of kinases through stabilizing the hydrophobic spine [21]. Other mutations-induced TKI resistances are relatively infrequent compared to gatekeeper mutations in FGFR, but they are still important (Table 2). For example, the FGFR1 N546K mutation confers resistance by increasing affinity for ATP [22]. The FGFR2 N550H mutation is regarded as an auto-inhibitory molecule brake that restricts the kinase to be an uncontrolled active state [23, 24]. The FGFR2 E565A mutation can up-regulate the PI3K/AKT/mTOR signaling pathway [25]. Besides, FGFR2 mutations are present in 12% of endometrial cancers [26], in which FGFR2 S252W is the most common mutation (9%) [27]. FGFR3 K650M mutation exists in 23.4% of FGFR mutated dedifferentiated liposarcomas, which predicts a poor prognosis [28].

**Table 2:** Mutations induced FGFR-TKIs resistance

TKI classification	TKI name	Mutation in kinase
Pan-FGFR inhibitors	TAS-120	FGFR2(V564F)
Multi-kinase inhibitors	TKI258	FGFR1(N546K); FGFR2(V564I,M536I,M538I,I548V,L618M,E719G,E565,K462,N550)
	Ponatinib	FGFR1(N546K); FGFR2(V564I); FGFR4(V550 E/L)
	E3810	FGFR1(V561M)
Selective FGFR inhibitors	AZD4547	FGFR1(V561M); FGFR2(I567, N568, V581, E584G, S587,K660E, K678M); FGFR3(V555M)
	BGJ398	FGFR2(N550H/K, V564F, E565A, K660M, L618V, K641R)
	PD173074	FGFR1(V561M, N546H); FGFR3(V555M)
	AZ12908010	FGFR3(V555M)
	Debio 1347	FGFR2(N550K, K660M, L618V)
	BLU-554	FGFR4(V550M/L, C552)

### FGFR1 gatekeeper mutation

Sohl and colleagues utilized structural and kinetic characteristics of FGFR1 to explain affinity changes for the FGFR inhibitors E3810 (Lucitanib) and AZD4547 due to the FGFR1 gatekeeper mutation V561M [29]. These studies showed that V561M mutation reduces affinity for E3810 (a double FGFR-VEGFR inhibitor), but that V561 mutant maintains nanomolar affinity for AZD4547 (a selective FGFR1-3 inhibitor) [29, 30]. Structurally, E3810 lacks flexibility, whereas the conformational flexibility of AZD4547 allows this TKI to adapt to the mutation [29, 31]. In addition, Sohl and colleagues used in vivo and in vitro binding assays to prove that the V561M mutant strongly activated STAT3 and produced significant resistance to AZD4547, thereby driving cancer progression [30]. By knocking out STAT3, cancer cells expressing FGFR1 V561M display restored sensitivity to AZD4547 [30]. These results suggest that knowledge of kinase-inhibitor complex structures alone is insufficient for a comprehensive understanding of the mechanisms of FGFR-TKI resistance. Downstream signaling pathways must also be considered.

### FGFR3 gatekeeper mutation

FGFR3 resembles FGFR1 structurally. The V561M gatekeeper mutation of FGFR1 corresponds to the V555M gatekeeper mutation of FGFR3. TKI258 (Dovitinib), a poly-kinase inhibitor can simultaneously retain its inhibitory effect in patients with FGFR1 and FGFR3 gatekeeper mutations, but the FGFR1 V561M mutant is markedly less sensitive to PD173074 and AZD4547 [32, 33]. Initially, patients with the FGFR3-TACC3 fusion are highly sensitive to AZD4547, but this TKI becomes ineffective after the appearance of FGFR3 gatekeeper mutations [32, 34]. Chell and his colleagues sequenced a KMS-11 myeloma cell line resistant to AZ12908010, and found the FGFR3 gatekeeper mutation V555M, which conferred cross-resistance to AZD4547 and PD173074 by an increase in FGFR3 phosphorylation and an improvement of downstream signaling transduction [34]. The molecular structural explanation is that the FGFR3 V555M mutation generates steric clashes with the phenyl ring of PD173074 by structural modeling, resulting in enhanced resistance [32, 34]. An additional factor is steric clashes caused by conformational changes in the P-loop region [22].

### FGFR2 gatekeeper mutation

In a clinical study of three patients with FGFR2-fusion cholangiocarcinoma receiving BGJ398 therapy, all patients developed acquired resistance with FGFR2 gatekeeper V564F mutation [35]. In the BaF3 cell line, which has high FGFR2 expression, researchers identified several Dovitinib-resistant mutations, including gatekeeper mutation [23]. Ponatinib (AP24534), a third-generation TKI, effectively inhibits all Dovitinib-resistant FGFR2 mutants except gatekeeper mutation [23]. Ponatinib, as a type II inhibitor, targets the inactive and DFG-out conformation, but can also target the active conformation [36].

Goyal et al. reported that the irreversible covalent FGFR inhibitor TAS-120 overcomes drug resistance induced by ATP-competitive FGFR inhibitors (such as BGJ398 and Debio 1347) in patients with FGFR2 fusion-positive intrahepatic cholangiocarcinoma [37]. Structurally, the FGFR2 gatekeeper V564F mutation induces steric clashes with the dichloro dimethoxy phenyl ring of BGJ398 [32, 35]. Debio 1347 generally retains activity against the FGFR2 V564F gatekeeper mutation, due to Debio 1347 partially replacing the large dimethoxyphenyl group with a benzimidazole moiety that makes stable contacts with V564F. Debio 1347 also confers resistance to other FGFR2 mutations [37, 38]. TAS-120 binds its target covalently, but it cannot resist FGFR2 gatekeeper mutations, due to steric clashes [37, 39].

### FGFR4 gatekeeper mutation

It is worth noting that mutations in FGFR4 are rare, but that Hatlen et al. identified gatekeeper mutations (V550M/L) and Hinge-1 (C552) mutations in the FGFR4 kinase domain from hepatocellular carcinoma patients after treatment with the selective FGFR4 inhibitor Fisogatinib (BLU-554) [40]. These mutants prevented Fisogatinib from covalently binding to FGFR4 [37]. Besides, FGFR4 V550M mutation was found in 13% of neuroendocrine breast cancers, and FGFR4 V550L mutation was found in 9% of embryonal rhabdomyosarcoma tumors [41, 42]. Huang et al. found that FGFR4 V550L mutant forms steric clashes with the imidazo[1,2-b]pyridazine scaffold of Ponatinib. Both preventing covalent binding and conflict formation reduced the inhibitory activity of TKI [43]. Surprisingly, our group found by in vivo and in vitro experiments that the Pan-FGFR inhibitor LY2874455 has a remarkable ability to overcome FGFR4 gatekeeper mutation-induced TKI resistance [44]. Further crystallographic experiment proved that LY2874455 binding site is distant from the gatekeeper residue, which avoids steric clashes with the ATP-binding pocket of FGFR4 [44, 45].

### Strategies for overcoming mutation-based FGFR-TKI resistance

**Fig. 1:**
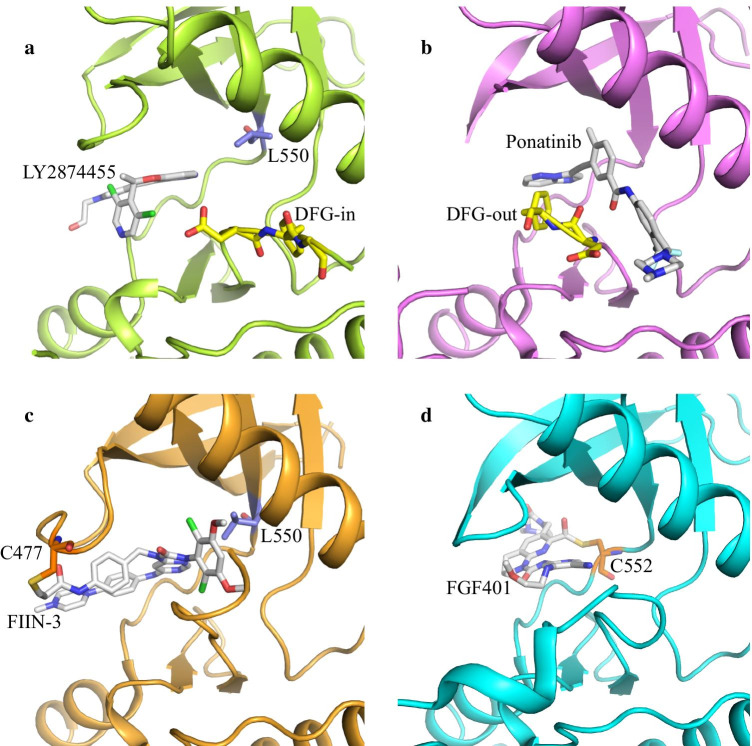
Strategies to overcome mutation-based FGFR-TKI resistance. **a** The binding site of LY2874455 is far away from gatekeeper residue L550 of FGFR4 (PDB: 5XFF). **b** Ponatinib binds to the FGFR kinase in an inactive and DFG-out conformation (PDB: 4V01). **c** FIIN-3 covalent binds the C477 of P-loop to overcome FGFR gatekeeper mutation-induced resistance (PDB: 4R6V). **d** FGF401 covalently binds residue C552 of Hinge to specific overcome the FGFR4 gatekeeper mutation (PDB: 6JPJ). Kinases are shown as cartoons, TKIs are shown in grey sticks, gatekeeper residues are shown in blue sticks, DFG motifs are shown in yellow sticks, and covalently bound cysteine is shown in brown sticks

When designing TKIs for FGFR gatekeeper mutations, we should take into account the following considerations: (1) The binding site for inhibitors can be far from the hinge region and, especially, from gatekeeper residues. For example, the pan-FGFR inhibitor LY2874455 is reported to be the most effective compound for all the different resistance mutations (Fig. 1a) [35, 40, 44]. The clinical phase I study showed that LY2874455 had nice tolerability and activity with an effective half-life of 12 h, weak toxicities, and RP2D (recommended phase 2 dosing) of 16 mg BID (bis in die) in patients with advanced solid-organ cancer [46]; (2) The inhibitors can selectively inhibit the active conformation of the kinase. Ponatinib binds to FGFR kinases in an inactive and DFG-out conformation (Fig. 1b) with IC50 of nanomolar in preclinical studies. And it is in phase II (NCT02272998) to treat advanced solid tumor patients with FGFR2-activated mutations [47]. However, Ponatinib is a multi-target kinase inhibitor that may cause strong side effects; (3) Cys-mediated covalent inhibitors can overcome gatekeeper mutations. FIIN-2 and FIIN-3 are the first covalent inhibitors based on the PD173074 scaffold. They can effectively bind covalently to C477 of the P-loop to overcome FGFR TKI resistance caused by FGFR gatekeeper mutations (Fig. 1c) [48]. Another case is FGF401, which covalently binds the C552 of Hinge to specifically overcome FGFR4 gatekeeper mutations (Fig. 1D) [49]. At present, FGF401 is in clinical phase I/II trials for hepatocellular carcinoma patients with FGF19/FGFR4 signal abnormity [50]. These covalent inhibitors with both selectivity and flexibility may be the main focus of targeted drug development in the future [51–53].

## Lysosome-mediated TKI sequestration reduces the kinase accessibility of TKI

**Fig. 2:**
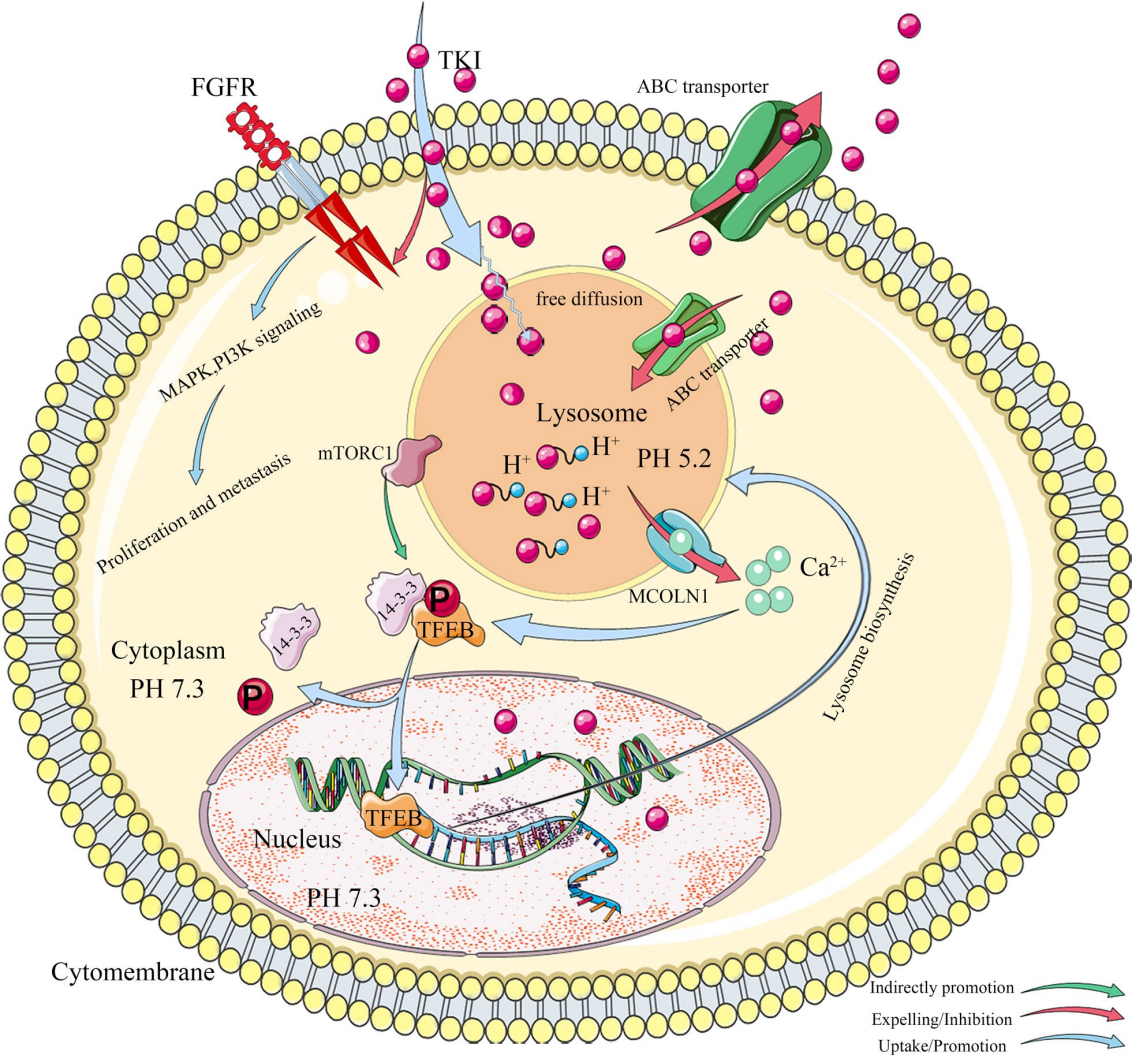
Mechanisms of lysosome-mediated FGFR-TKI sequestration. Firstly, lipophilic weakly basic TKIs are trapped in the lysosome cavity by free diffusion and protonation. Secondly, TKIs are pumped into lysosome by ABC transporters. Thirdly, under stimulation of mTORC1 inactivation and TKI-induced Ca^2+^ release, the transcription factor TFEB translocates into the nucleus and mediates lysosome biosynthesis, enhancing TKI sequestration

Computer-aided drug models and the application of structural biology can reveal interactions between drugs and their targets in vitro. However, kinetic changes of anticancer drugs in cells can also affect their efficacy, and even cause drug resistance. Lysosomes are digestive vesicles composed of a lipoprotein membrane. They contain various acidic hydrolases that eliminate excess proteins, nucleic acids, lipids, polysaccharides, and other macromolecules in the cell [54–56]. It is also an ideal container for sequestration of weakly base TKIs away from their targets, which changes the kinetics of TKIs and results in drug resistance. Lysosomes mediate TKI sequestration in the following ways (Fig. 2).

### Lipophilic weakly basis TKIs are sequestrated into lysosome by diffusion

After lipophilic weakly basic TKIs enter the cell, they can diffuse freely into lysosomes, driven by the pH gradient change between lysosomes and cytoplasm. The drug is then protonated in an acidic environment and cannot re-cross the lysosomal membrane. It is sequestrated in the lysosomal vesicle, which prevents the drug from reaching its target, resulting in a decrease in drug concentration and drug resistance [55]. Sunitinib and Gefitinib (a selective EGFR-TKI) were reported to have a significant sequestration effect in lysosomes [55, 57].

Englinger and colleagues have documented lysosome-induced drug sequestration in lung cancer with FGFR changes. They analyzed the cell-free fluorescence, intracellular accumulation, and distribution of the multi-kinase inhibitor Nintedanib and the FGFR kinase inhibitor PD173074 using three-dimensional fluorescence spectroscopy, together with analytical chemistry and molecular biology methods. These studies revealed selective accumulation of drugs in the lysosome, and verified the lipophilic and weakly basic drug characteristics of Nintedanib and PD173074 [58–60]. However, this mechanism is reversible and has a less stable genetic resistance effect [61]. Targeting lysosomes in tumors therapy can be a promising strategy to overcome drug resistance. Besides, autophagy is closely related to lysosome-mediated TKI resistance [62]. Rui Peng and colleagues found that activation of the AMPK/mTOR signaling pathway induced survival autophagy to resist drug therapy in FGFR-TKI resistant gastric cancer cell lines. And TAK1 (TGF-β-activated kinase 1) over-expression enhanced the activation of AMPK signaling and autophagy. Further in vitro and in vivo studies demonstrated that the TAK1 inhibitor NG25 and FGFR inhibitor AZD4547 synergistically inhibited proliferation and autophagy in AZD4547-resistant cell lines and patient-derived xenograft tumor model [63]. It suggests that TAK1 inhibitors cooperated with FGFR inhibitors may be a new therapeutic strategy to overcome autophagy-mediated FGFR-TKI resistance.

### ABC transporters pump TKI into the lysosome

ATP-binging cassette (ABC) superfamily transporters are located on cytoplasmic and lysosomal membranes. Cytoplasmic ABC transporters pump TKIs out of the cytoplasm. Lysosomal ABC transporters pump TKI into lysosomes. This process promotes the sequestration of TKIs in lysosomes [56, 64]. For example, ABCG2 can mediate active transport of Imatinib into lysosomes [65]. Moreover, ABC transporters can cooperate with lysosome sequestration to aggravate drug resistance [66].

### TFEB-mediated lysosome biosynthesis enhances TKI sequestration

TFEB, as a key transcription factor, regulates the biogenesis of lysosomes. Under physiological conditions, Ser142 of TFEB is phosphorylated by lysosomal mammalian target rapamycin complex 1 (mTORC1), and the phosphorylated TFEB is retained in the cytoplasm by forming a complex with YWHA (14-3-3) protein, resulting in inactive transcription. In contrast, lysosome-sequestrated TKI is protonated in an acidic environment, which increases the permeability of lysosomal membrane via fluidization and inhibits the activity of mTORC1 kinase. The high permeability of the lysosomal membrane increases the release of Ca^2+^ into cytoplasm to activate calcineurin, which further dephosphorylates TFEB. Dephosphorylated TFEB then dissociates from the 14-3-3/TFEB complex, and enters into the nucleus to initiate transcription of lysosome-associated proteins. In this way, TFEB promotes lysosome generation and increases TKI sequestration and resistance [67–69].

### Intervention strategies for lysosome sequestration of TKI

The most direct way to prevent lysosome sequestration-induced drug resistance is to change the structure of the TKI [56], such as by assembling the TKI onto nanoparticles for delivery into the cell [70]. Another way is targeting the lysosome to eliminate lysosome-mediated TKI sequestration [56, 71]. This approach can involve: (1) Targeting the H + ATP enzyme (maintaining lysosome acidity) to alkalize lysosomes; (2) Lysosomotropic agents, like chloroquine, disturb lysosomal sequestration of TKIs by inhibiting autophagy or de-acidification [56, 66]; (3) Imidazoacridones (IAS) can cause lysosomal photodestruction, which destroys the internal structure of lysosomes [72]; (4) Acid sphingomyelinase (ASM) inhibitors and lysosomal membrane protein (LMP) inhibitors can destroy the stability of the lysosomal membrane and alter membrane permeability [59], which is essential to maintain the pH gradient between lysosome and cytoplasm. Blocking TFEB-mediated lysosomal generation should also be an effective strategy to reduce FGFR-TKI sequestration. Lastly, we can combine kinase inhibitors with autophagy inhibitors and ABC transporter-related inhibitors to overcome resistance. For example, ABCB1 has been reported to be a key player in resistance to the multiple-target kinase inhibitor Nintedanib [73, 74]. Inhibition of ABCB1 and kinases simultaneously should be a promising strategy for overcoming resistance [75–77].

## Alternatively activated signaling pathways bypass FGFR inhibition

**Fig. 3:**
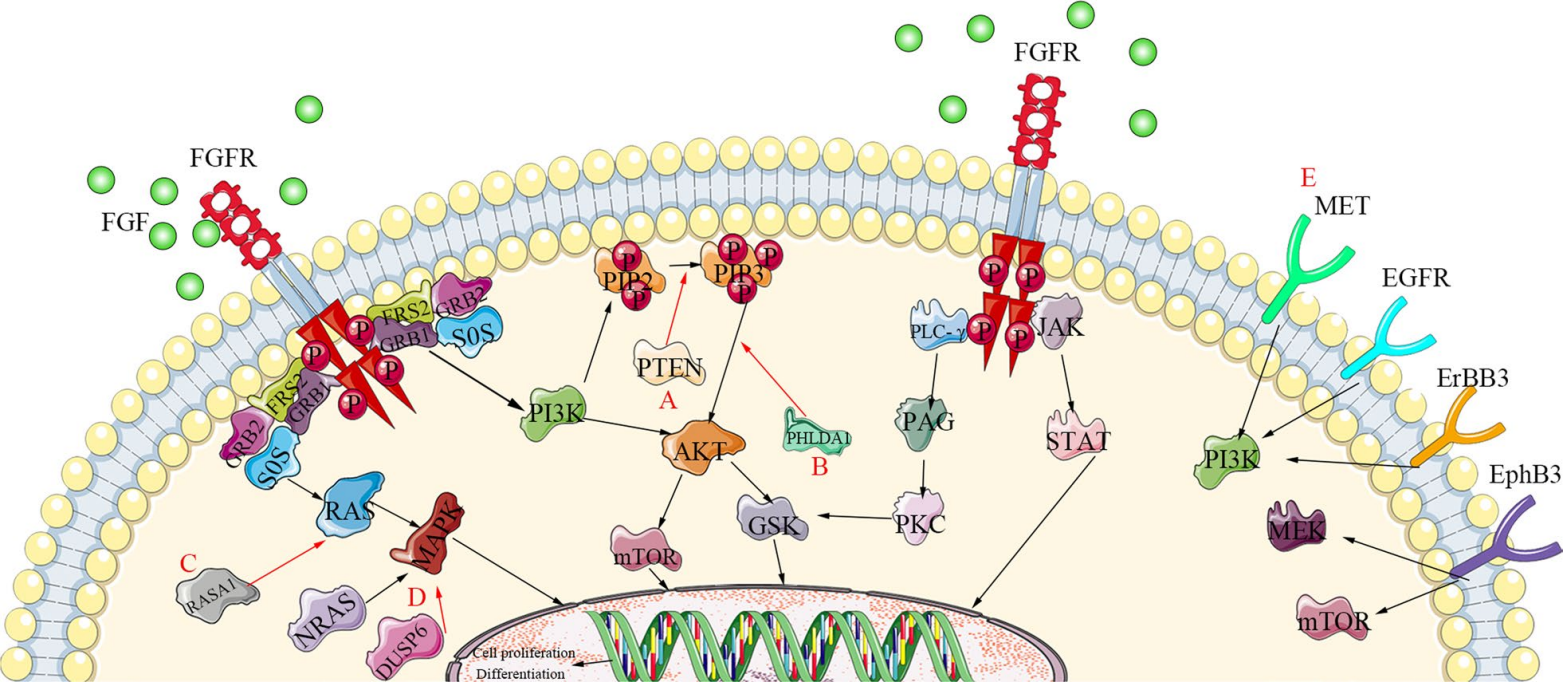
Mechanisms of alternative signaling activation induced FGFR-TKI resistance. FGFR signaling pathway regulation networks are shown in black arrows. Mechanisms of FGFR-TKI resistance caused by feedback signaling activation are shown. **a** Loss of PTEN up-regulates the expression of PIK kinase via interfering with the conversion of PIP2 to PIP3 and abortively activates AKT; **b** inactivation of PHLDA1 continuously activates AKT signaling by competitively binding AKT with PIP3; **c** inactivation of RASA1 directly activates RAS-MAPK pathway through regulating the active transformation of RAS; **d** NRAS amplification and DUSP6 deletion stimulate activation of the MAPK pathway; **e** alternative activation of cytomembrane localized kinases, such as EGFR, MET and ErbB3, bypass FGFR signaling pathway

FGFR-TKI resistance mediated by signaling pathways involves alternative activation of two downstream branches of the FGFR signal (PI3K-AKT and RAS-MAPK) and other membrane RTKs pathways (Fig. 3).

### PI3K-AKT activation

Secondary activation of the PI3K-AKT signaling pathway is a classical mechanism for FGFR-TKI resistance [78]. Jharna Datta and colleagues found that the phosphorylation levels of AKT and downstream GSK3 were up-regulated in BGJ398 resistant DMS114 (FGFR1-amplificated SCLC) and RT112 (urothelial carcinoma with FGFR aberrations) cell lines. And the drug-resistant cell lines recovered their sensitivity to BGJ398 after blocking the PI3K-AKT signal by AKT inhibitor GSK2141795 or siRNA intervention [79].

The PI3K-AKT signaling pathway can be directly regulated by Pleckstrin Homology-Like Domain family A member 1 (PHLDA1) and Phosphatase and tensin homolog (PTEN), resulting in TKI-resistance (Fig. 3) [78]. PHLDA1 can competitively bind PIP3 with AKT to inhibit the activity of AKT. Once PHLDA1 is knocked down, the continuous activation of AKT signaling is sufficient to sustain cell proliferation and survival, which can induce new resistance to FGFR inhibitors PD173074 and AZD4547 in endometrial cancer cells [80]. PTEN belongs to the protein tyrosine phosphatases (PTP) gene family. Knockout of PTEN can enhance the phosphorylation of AKT, and up-regulate the expression of PIK. It suggests that PTEN deletion is a potential mechanism to resist FGFR inhibition in endometrial cancer cells [81–83]. Besides, GSK3, as a downstream molecule of PI3K-AKT signaling, can also be activated by PKC signal in an AKT-independent manner, leading to resistance to FGFR inhibitor AZD4547 in Diffuse-Type Gastric Cancer [84].

### RAS-MAPK activation

Increased activation of the RAS-MAPK pathway plays a critical role in FGFR-TKI resistance [78, 85] Kas and colleagues showed that Ras p21 protein activator 1 (RASA1) is a negative regulator of RAS, whose inactivation directly causes activation of the RAS-MAPK pathway [86]. For instance, knockdown of RASA1 activated downstream signaling of RAS, promoting cell growth and causing resistance to FGFR inhibition. In contrast, the recovery of RASA1 expression in RASA1-mutated cells reduced MAPK and PI3K signaling pathways [86, 87].

In addition, as positive and negative factors in regulating MAPK signaling pathways, abnormalities of NRAS and DUSP6 may affect FGFR-TKI resistance. For example, in drug-resistant lung cancer cells, NRAS amplification [88] and DUSP6 deletion lead to a reactivation of the MAPK pathway, thereby resisting FGFR inhibitors [89]. Co-inhibition of FGFR and MAPK pathway by FGFR inhibitors and MEK inhibitor Trametinib induced tumor degradation in tumor xenografts derived from mesenchymal-like KRAS mutant cancer cell lines as well as patient-derived xenograft model with a typical mesenchymal phenotype [90].

### Membrane RTKs activation

Alternative activation of membrane RTKs, such as ErbB3 [91, 92], MET [93, 94], EGFR [95], EphB3 [96, 97], KIT [98], and the crosstalk between RTK and FGFR, account for the resistance of FGFR targeted therapies [99, 100]. A functional genetic screen has found that feedback activation of ErbB3 (Erb-B2 receptor tyrosine kinase 3) signaling pathway can reduce the sensitivity of AZD4547 through activation of downstream PI3K pathway in urothelial carcinoma [91]. Notably, ligand-mediated activation of ErbB2/3 can directly lead to BGJ398 resistance in FGFR3-dependent cancer cells [92].

Singleton et al. found that the activation of MET plays a crucial role in resistance to the FGFR inhibitor AZ8010 [93]. Smurm Kim et al. proved that the enhanced MET can activate PI3K/AKT signaling pathway in an ErbB3-dependent or independent manner to obtain resistance to FGFR-TKIs [94]. Moreover, MET and FGFR can compensate for each other by regulating the activation of downstream signaling pathways [101].

Using parallel RNA interference genetic screens, Maria and colleagues demonstrated that the activation of EGFR can limit the sensitivity of PD170374 in bladder cancer. Combination of FGFR and EGFR inhibitors by PD173074 and Gefitinib overcome this resistance in vitro and in vivo, but with poor tolerance in mice [95]. On the contrary, activation of the FGFR signaling pathway also associates with resistance to EGFR inhibitors [102].

Lee et al. reported that the EphB3 signaling pathway was alternatively activated in FGFR inhibitor AZD4547 resistant gastric cancer cell line SNU-16R, thereby promoting epithelial-to-mesenchymal transition (EMT) of gastric cancer cell through activation of Ras-ERK1/2-mTOR pathway [96, 97]. Blocking EphB3 by LDN-211904 reduced the phosphorylation of the Ras-ERK1/2-mTOR signal and inhibited EMT in SNU-16R cells [96]. The reactivation of mTOR signaling also reduced the sensitivity of FGFR2-amplified tumors to AZD4547 [96].

Bauer and colleagues found that KIT activation was 3–6 folds higher in GIST430 and GIST48 (Imatinib-resistant gastrointestinal stromal tumor) than in GIST882 (Imatinib-sensitive) [98]. And targeting downstream signaling molecular PI3K resulted in substantial apoptosis in the Imatinib-resistant gastrointestinal stromal tumor [98]. Besides, signaling crosstalk between KIT and FGFR3 activated the MAPK pathway to promote resistance to Imatinib. Co-inhibition of KIT and FGFR3 synergistically blocked the growth of Imatinib-resistant cells [103].

### Strategies for blocking alternatively activated signaling

To overcome drug resistance, maintaining a high response rate to kinase inhibitors is essential. One approach employs combination therapy, which can block multiple activation pathways simultaneously [104]. For example, in ovarian cancer xenografted mice, co-inhibition of FGFR and mTOR pathway simultaneously by BGJ398 and rapamycin-induced tumor regression, cell cycle arrest, and apoptosis [105]. Intriguingly, FGFR2-TACC3 fusion protein identified in cholangiocarcinoma appears to be a client of heat shock protein 90 (HSP90). The HSP90 inhibitor Ganetespib combined with BGJ398 can greatly inhibit signaling transduction of FGFR2-TACC3 fusion protein [106]. In addition, combination therapy with immune checkpoint inhibitors and combination endocrine therapy with hormonal changes in cancer patients have been reported [107, 108]. For example, co-inhibition of FGFR and PD-1 by Erdafitinib and Cetrelimab (PD-1 targeted monoclonal antibody) is in phase Ib /phase II (NCT03473743) against previously untreated cisplatin-ineligible patients [109].

Combination therapy has great promise, but it may elicit undesirable drug–drug interactions. Therefore, another approach is rationally designing single compounds with dual targets [110]. As a pan-inhibitor of FGFR1-3, 3D185 not only inhibits FGFR in tumor cells but also target CSF-1R (the main survival factor of macrophages), which is vital to the immunosuppressive microenvironment [111]. Another dual-targeted inhibitor is MPT0L145, which targets PIK3C3 and FGFR simultaneously [112]. These double active inhibitors provide new approaches to drug design.

## Gene fusion enhances the activation of downstream signaling

The term gene fusion refers to gene rearrangements caused by chromosome inversion, interstitial deletion, repetition, or translocation, which are prevalent in various cancers [113]. Oncogene fusions can constitutionally activate tyrosine kinases and enhance downstream survival signaling. Hence, gene fusions have a significant effect on the development of many solid tumors [113].

### FGFR gene fusions directly account for FGFR-TKI resistance

FGFR-TKI resistance events can be directly triggered by FGFR gene fusion [114]. Kim and colleagues identified a novel FGFR2-ACSL5 fusion from a metastatic gastric cancer patient with FGFR2 amplification through RNA sequencing [115]. Intriguingly, at the beginning of FGFR inhibitor treatment, the patient showed strong sensitivity to LY2874455, and no FGFR2-ACSL5 fusion was found in vivo, but eventually, drug resistance was detected along with the FGFR2-ACSL5 fusion gene [115]. Additionally, PIK3-AKT-mTOR pathways were greatly activated in gene fusion- expressing cell lines [115]. However, this study represents an individual case and is not generalized. The function of FGFR2-ASCL5 fusion proteins is not yet clear and maybe affected by tumor heterogeneity and body environment.

### JHDM1D-BRAF gene fusion indirectly induces FGFR-TKI resistance

The occurrence of FGFR-TKI resistance events can also be triggered by other related gene fusion. Sase et al. performed a study on the mechanism of resistance to FGFR small molecule inhibitors in FGFR2-amplified gastric cancer. They found that the fusion kinase JHDM1D-BRAF located on chromosome 7 confers resistance to AZD4547 in a monoclonal gastric cancer cell line SUN-16. Jumonji C domain-containing histone demethylase 1 homolog D (JHDM1D) is a histone demethylase that plays a major role in neural differentiation [116]. BRAF, which regulates the MAPK/ERK signaling pathway, encodes the RAF family of serine/threonine protein kinases [117]. After JHDM1D-BRAF fusion, constructive dimerization of the fusion protein was enhanced, accompanied by activation of the downstream MAPK pathway, the disappearance of FGFR2 phosphorylation, and a decrease in FGFR2 expression in SUN-16 cells. These results suggest that co-treatment of RAF dimer inhibitors with downstream signaling molecule MEK inhibitors may be an option for avoiding resistance [118] (Table 3).

**Table 3:** The mechanisms of FGFR-TKIs resistance

TKI classification	TKI name	Involved mechanisms
Pan-FGFR inhibitors	LY2874455	FGFR2-ACSL5 fusion
Multi-kinase inhibitors	Ponatinib	PTEN
	Nintedanib	ABCB1 induced drug-efflux; Lysosomal sequestration
Selective FGFR inhibitors	AZD4547	Gene fusion: JHDM1D-BRAF fusion; Alternative pathways activation: RAS-MAPK pathway, ErbB3/PI3K/AKT pathway, MET; Related molecular abnormalities: RASA1, PHLDA1, PTEN, STAT3; Other: EMT; Drugs-efflux
	BGJ398	Alternative pathways activation: RAS-MAPK pathway, PI3K/AKT /GSK pathway, MET, ErbB2/3 pathway; Related molecular abnormalities: NRAS, DUSP6; Other: EMT
	PD173074	EGFR signaling pathway; PHLDA1; EMT; Lysosomal sequestration
	AZ12908010	MET

## Conclusions

With the increase in FGFR inhibitors undergoing clinical or pre-clinical trials, the resistance to FGFR-TKIs has become a major issue. The main mechanisms responsible for resistance can be summarized as follows: (1) Gatekeeper mutation-induced steric clashes that interfere with TKI binding; (2) Feedback activation of alternative signaling pathways that bypass FGFR inhibition; (3) Lysosome sequestration-mediated TKI “kidnapping” that promotes TKI retention in lysosomes and prevents TKIs from reaching their target kinases; (4) Gene fusions that induce continuous activation of downstream signaling, thereby eliminating the inhibition of TKI.

In addition, the acquired resistance mediated by FGF-FGFR signal axis should be considered. (1) Activation of the FGF2-FGFR1 autocrine pathway results in acquired resistance to Gefitinib in NSCLC [119, 120]. (2) Nuclear translocation of FGF2/FGFR1 facilitates pancreatic cancer cell invasion, leading to TKIs resistance [121]. (3) FGFR1 amplification induces receptor accumulation and continuously activates the downstream signaling pathways [122], and (4) abnormally up-regulated FGFR ligands disrupt the autocrine loop of growth factor, leading to lung cancer resistance to TKIs [120].

Therapeutic strategies for overcoming FGFR TKI resistance mainly center on four approaches: (1) Developing new FGFR TKIs, especially covalent inhibitors, to specifically overcome mutation-induced TKI resistance; (2) Adopting combination therapies that target multiple pathways simultaneously; (3) Disrupting the architecture of lysosomes to release sequestrated TKI; and (4) Exploiting FGFR ligand or FGFR specific monoclonal antibodies to bypass TKI resistance [107, 123].

The potential strategies to predict and bypass TKI resistance can prospect from the following three aspects: (1) Developing computer machine learning algorithm by multivariable models to predict TKI resistance factors for early diagnosis and intervention of patients with drug resistance, which have achieved big progress on individualized therapy of patients with acquired EGFR-TKI resistance [124]; (2) Exploiting Physics-Based and Data-Driven Approaches to screen inhibitors through predicting affinity changes between TKIs and kinases based on kinase mutations, reducing the occurrence of TKI resistance [125, 126]; (3) Adopting next-generation sequencing (such as ctDNA sequencing), molecular detection, and tumor biopsy detection of FGFR gene abnormalities in patients to predict/bypass the occurrence of TKI resistance and evaluate appropriate treatment regimen [127, 128]. In a word, the ultimate goal of FGFR-TKI targeted therapy should be to move toward precision medicine and individualized treatment to develop optimal treatment strategies.

## Data Availability

The materials supporting the conclusion of this review have been included in the article.

## Abbreviations

FGFR: Fibroblast growth factor receptor
FGF: Fibroblast growth factor
TKI: Tyrosine kinase inhibitor
STAT3: Signal transducer and activator of transcription
TACC3: Transforming acidic coiled-coil containing protein3
PI3K: Phosphatidylinositol 3-kinase
AKT: AKT serine/threonine kinase
mTOR: Mechanistic target of rapamycin kinase
PHLDA1: Protein Pleckstrin Homology-Like Domain, family A, member 1
PTEN: Phosphatase and tensin homolog
PTP: Protein tyrosine phosphatases
PIP3: Plasma membrane intrinsic protein 3
GS3Kβ: Glycogen synthase kinase 3 beta
PKC: Protein kinase C
ErbB3: Erb-B2 receptor tyrosine kinase 3
EGFR: Epidermal growth factor receptor
RTK: Receptor tyrosine kinase
KIT: KIT proto-oncogene, receptor tyrosine kinase
MAPK: Mitogen-activated kinase-like protein
EMT: Epithelial mesenchymal transition
MET: MET proto-oncogene, receptor tyrosine kinase
RASA1: Ras p21 protein activator 1
EphB3: Eph receptor B3
MEK: MAP kinase–ERK kinase
VEGFR: Vascular endothelial growth factor receptor
ABC: ATP-binging cassette
TFEB: Transcription factor EB
mTORC1: Mammalian target rapamycin complex 1
IAS: Imidazoacridones
ASM: Acid sphingomyelinase
LMP: Lysosomal membrane protein
ACSL5: Acyl-CoA synthetase long chain family member 5
JHDM1D: Jumonji C domain containing histone demethylase 1 homolog D
BRAF: B-Raf proto-oncogene
TAK1: TGF-β-activated kinase 1
RP2D: Recommended phase 2 dosing
BID: Bis in die
AMPK: AMP-activated protein kinase
ORR: Objective response rate
DCR: Disease control rate
PFS: Progression-free survival
OS: Overall survival
DOR: Duration of response
CBR: Clinical benefit rate
